# Aqueous extract of *Psidium glaziovianum* Kiaersk leaves acts on muscarinic and opioid receptors and reduces cytokines and inflammatory infiltrates in vivo models

**DOI:** 10.1007/s10787-026-02250-1

**Published:** 2026-04-25

**Authors:** Wêndeo Kennedy Costa, Paulo Henrique Eloi Fernandes, Paloma Maria da Silva, Samuel José Oliveira de Freitas, Eduardo Alves Camêlo, Gildomar Guilherme Interaminense Nunes, Janaina Carla Barbosa Machado, Magda Rhayanny Assunção Ferreira, Luiz Alberto Lira Soares, Alisson Macário de Oliveirad, Márcia Vanusa da Silva, Maria Tereza dos Santos Correia

**Affiliations:** 1 Departamento de Bioquímica, Universidade Federal de Pernambuco, Recife, PE 50670-901 Brazil; 2 Departamento de Ciências Farmacêuticas, Universidade Federal de Pernambuco, Recife, PE 50670-901 Brazil; 3 Departamento de Engenharia Biomédica, Universidade Federal de Pernambuco, Recife, PE 50670-901 Brazil; 4 Universidade Federal do Rio Grande do Norte, Natal, RN 59078-900 Brazil

**Keywords:** Analgesic activity, Cytokines, Inflammation, Opioid pathway, Muscarinic receptor

## Abstract

Pain and inflammation are essential physiological processes, yet their dysregulation contributes to chronic pathological conditions. Conventional analgesic and anti-inflammatory therapies often cause adverse effects, highlighting the need for safer alternatives. *Psidium glaziovianum* (Myrtaceae) is a medicinal species rich in bioactive compounds with potential pharmacological activity. This study evaluated the analgesic and anti-inflammatory properties of the aqueous extract of *P. glaziovianum* leaves (EAPg) in experimental models of nociception and inflammation. Chromatographic and UV-Vis analyses revealed the presence of flavonoids and ellagic acid. EAPg induced a dose-dependent antinociceptive effect, with the 100 mg/kg dose being the most effective in reducing nociceptive behaviors in acetic acid and formalin tests and increasing latency in the tail immersion test, suggesting both peripheral and central actions. Pretreatment with naloxone and atropine partially reversed the antinociceptive effects, indicating the participation of opioid and muscarinic receptors. In inflammatory models, EAPg significantly reduced paw edema and leukocyte recruitment and decreased TNF-α and IL-1β levels, demonstrating potent anti-inflammatory activity. Collectively, these findings provide the first evidence that EAPg exerts dual (central and peripheral) antinociceptive and anti-inflammatory effects mediated through opioid and muscarinic pathways, supporting its pharmacological potential as a natural analgesic and anti-inflammatory agent.

## Introduction

Pain and inflammation are interconnected physiological responses that play essential roles in defending the body against injury, infection, and other harmful stimuli. Pain functions as a warning signal of actual or impending tissue damage, triggering protective reflexes and promoting withdrawal from harmful agents. Inflammation, in turn, is a coordinated process involving activation of the immune system, release of chemical mediators, increased vascular permeability, and recruitment of immune cells to the affected site, thereby facilitating tissue repair and restoration of homeostasis (Hong et al. 2023; Dainese et al. 2023; Knights et al. 2023).

However, when these responses become exaggerated, dysregulated, or persistent, they lose their protective nature and significantly contribute to the development of chronic diseases such as rheumatoid arthritis, inflammatory bowel diseases, neuropathic pain, fibromyalgia, and even certain types of cancer. In these contexts, both pain and inflammation become dysfunctional, with molecular and cellular mechanisms sustaining a state of constant activation, leading to nociceptor sensitization, enhanced neuronal excitability, and continuous release of pro-inflammatory cytokines (Kou et al. 2023; Bruner et al. 2023; Di Matteo et al. 2023; Chang et al. 2024; Ashton and Beattie 2024).

This condition negatively impacts patients’ quality of life and poses a significant therapeutic challenge, as pharmacological management of pain and inflammation typically relies on analgesics and non-steroidal anti-inflammatory drugs (NSAIDs). Despite their efficacy, these drugs are frequently associated with adverse effects such as gastrointestinal disorders, nephrotoxicity, and tolerance with prolonged use (Minhas et al. 2023; LaForge et al. 2023). Consequently, there is growing interest in safe and effective therapeutic alternatives, particularly those derived from medicinal plants (Costa et al. 2022).

Brazilian plant biodiversity has proven to be a promising source of bioactive compounds with relevant pharmacological properties (Zhang et al. 2021; Costa et al. 2022; Shady et al. 2022). Among the species with therapeutic potential, *Psidium glaziovianum* Kiaersk, commonly known as araçá-rosa and belonging to the Myrtaceae family, stands out. Recent studies have demonstrated that its essential oil exhibits analgesic, anti-inflammatory, and wound-healing activities. Nevertheless, scientific evidence regarding the pharmacological potential of its extracts and the mechanisms underlying its analgesic and anti-inflammatory effects remains scarce (Costa et al. 2022, 2023a; Costa et al. 2023b; Costa et al. 2024).

In this context, the present study aimed to investigate the antinociceptive and anti-inflammatory effects of the aqueous extract of *P. glaziovianum* leaves in in vivo experimental models. In addition to assessing pharmacological efficacy, we sought to elucidate the mechanisms underlying these effects.

## Materials and methods

### Plant material collection and preparation

Leaves of *P. glaziovianum* were collected on October 17, 2019, in the municipality of Exu, Pernambuco State, Brazil (7° 21′ 16.3′′ S; 39° 53′ 15.5′′ W). Botanical identification was performed, and a voucher specimen was deposited under number 93,728 at the Dárdano de Andrade Lima Herbarium, Agronomic Institute of Pernambuco (IPA/PE). The leaves were dried in a forced-air circulation oven (Lucadema^®^) for 48 h at 45 °C. After drying, the material was ground in a knife mill (Tecnal^®^). The extract was prepared by turbolysis using distilled water at a 10% (w/v) ratio, with three 30-second cycles interspersed with 4-minute pauses. The material was then concentrated using a rotary evaporator and subsequently lyophilized, yielding a powdered aqueous extract referred to as AEPg. The yield of the extraction was calculated based on the dry weight of the plant and expressed as a percentage, resulting in a yield of 1.02% (w/w).

### Phytochemical analysis

An aliquot of 5 mg of the extract was weighed and transferred to a 10 mL volumetric flask, solubilized in 50% ethanol (v/v), and filtered into vials using PVDF filters (0.45 μm). HPLC analysis was performed using an Ultimate 3000 system (Thermo Fisher Scientific, USA) equipped with a diode array detector (DAD; Thermo Fisher Scientific), a binary pump (HPG-3 × 00RS, Thermo Fisher Scientific), a degasser, and an autosampler fitted with a 20 µL loop (ACC-3000, Thermo Fisher Scientific). The detection wavelength was set at 270 nm.

Chromatographic separation was achieved on a Dionex^®^ C18 column (250 mm × 4.6 mm i.d., 5 μm) equipped with a guard column (C18, 4 mm × 3.9 mm; Phenomenex^®^). The column temperature was maintained at 27 ± 1 °C. The mobile phase consisted of ultrapure water (A) and methanol (B), both acidified with 0.05% trifluoroacetic acid, at a flow rate of 0.7 mL/min. A gradient program was applied as follows: 0–10 min, 15–25% B; 10–15 min, 25–40% B; 15–20 min, 40–80% B; 20–25 min, 80% B; 25–28 min, 80–40% B; 28–30 min, 40–15% B. Analyses were performed in triplicate, and data were processed using Chromeleon 6.8 software (Dionex/Thermo Fisher Scientific, USA).

### Ethical approval

All animal experiments were conducted in compliance with Brazilian guidelines for research ethics and were approved by the Ethics Committee on Animal Use of the Federal University of Pernambuco (protocol no. 122/2019). Male Swiss *Mus musculus* mice (30–40 g, 60 days old) were obtained from the Keizo Asami Immunopathology Laboratory (LIKA, UFPE). Animals were acclimatized for two weeks under controlled conditions (24 °C, 12 h light/dark cycle) at the Animal Experimentation Laboratory, Department of Biochemistry, UFPE. Prior to the procedures, animals were fasted for 6 h with free access to water.

The number of animals per group (*n* = 6) was determined based on previous studies from our group and the literature employing similar nociception and inflammation models, which consistently demonstrate that this sample size is sufficient to detect pharmacologically relevant differences using ANOVA-based statistical analysis. Historical variability data from our laboratory were also considered. The sample size was defined in accordance with the 3Rs principles (Replacement, Reduction, and Refinement), ensuring adequate statistical power while minimizing animal use. All experimental procedures were conducted using coded treatment allocation to minimize potential bias. The investigator responsible for assessments was blinded to group allocation during data collection and analysis. Treatment codes were only revealed after completion of statistical analysis.

### Evaluation of antinociceptive activity

Mice were randomly assigned to six groups (*n* = 6): Group I received saline solution (0.9%, p.o.); Groups II, III, and IV were treated with aqueous extract of *Psidium glaziovianum* (AEPg) at doses of 25, 50, and 100 mg/kg, respectively (p.o.); Group V received morphine (10 mg/kg, i.p.); and Group VI received indomethacin (20 mg/kg, i.p.). AEPg and saline were administered 60 min before the beginning of the tests, whereas reference drugs were given 30 min before testing.

#### Acetic acid-induced writhing test

The test was conducted according to the method described by Oliveira et al. (2018). Pain was induced by intraperitoneal injection of 0.85% (v/v) acetic acid at a volume of 0.1 mL/10 g body weight. Mice were then placed in observation chambers, and the number of abdominal writhes was recorded between 5 and 15 min after injection.

#### Formalin test

Each mouse received 20 µL of 2.5% (v/v) formalin injected into the subplantar region of the right hind paw. The time spent licking the paw was recorded in two distinct phases: 0–5 min (neurogenic phase) and 15–30 min (inflammatory phase), following the protocol described by Hunskaar and Hole (1987).

#### Tail immersion test

Twenty-four hours before the experiment, mice underwent a screening test in water maintained at 55 ± 1 °C. Animals that kept their tails immersed for more than 5 s were excluded from the study. Eligible mice were then allocated into groups (*n* = 6) and treated according to the experimental protocol. The latency time for nociceptive response was recorded at 0, 30, 60, 90, and 120 min after treatment, with a cut-off time of 20 s, as described by Khatun et al. (2015).

#### Investigation of mechanisms underlying the antinociceptive activity

To identify the mechanisms responsible for the analgesic effect of AEPg, mice were pretreated with naloxone (2 mg/kg, i.p.), atropine (1 mg/kg, i.p.), glibenclamide (5 mg/kg, i.p.), prazosin (1 mg/kg, i.p.), or yohimbine (1 mg/kg, i.p.). Fifteen minutes later, animals received AEPg (100 mg/kg, p.o.), saline (0.9%, p.o.), or morphine (10 mg/kg, i.p.) and were evaluated using the formalin test.

### Evaluation of anti-inflammatory activity

The anti-inflammatory property of AEPg was investigated using carrageenan-induced paw edema and peritonitis models. In all experiments, mice were randomly divided into five groups (*n* = 6): Group I received saline solution (0.9%, p.o.); Groups II–IV were treated with AEPg at doses of 25, 50, or 100 mg/kg (p.o.); and Group V received indomethacin (20 mg/kg, i.p.). AEPg and saline were administered 60 min before induction of the experimental models, whereas indomethacin was administered 30 min prior, according to Oliveira et al. (2018).

#### Carrageenan-induced paw edema model

Paw edema was induced by subplantar injection of 15 µL of 2% carrageenan solution in the right hind paw, a modification of the original method by Winter et al. (1962), previously optimized to reduce animal discomfort while ensuring reproducible inflammatory response. Paw volume was measured using a digital caliper before induction and at 1, 2, 3, and 4 h after injection (Lapa et al. 2008; Andrade et al. 2024).

#### Carrageenan-induced peritonitis model

Peritonitis was induced by intraperitoneal injection of 1% carrageenan (100 µL/10 g body weight). Four hours later, mice were euthanized, and peritoneal exudate was collected by lavage with 2 mL PBS containing heparin. Total leukocyte counts were determined using a hematology analyzer, with results expressed as a percentage relative to the control group (Lapa 2008). In addition, concentrations of the pro-inflammatory cytokines TNF-α and IL-1β in the exudate were quantified according to the method described by Oliveira et al. (2018).

### Statistical analysis

Data were analyzed using GraphPad Prism^®^ version 8.0 and expressed as mean ± standard deviation. Statistical analysis was performed using ANOVA followed by Dunnett’s post hoc tests, as appropriate. Differences were considered statistically significant at *p* < 0.001 in the antinociceptive and anti-inflammatory assays.

## Results and discussion

In the aqueous extract of *P. glaziovianum* leaves, peaks corresponding to the presence of flavonoids were detected (absorption maxima at 202, 258, and 354 nm, F1 and F2). These peaks did not match the retention times of the injected flavonoid standards (rutin and quercetin), although their scan spectra were consistent with the flavonoid class. In addition, the presence of ellagic acid (absorption maxima at 254 and 356 nm) and an ellagic acid derivative (absorption maxima at 249 and 360 nm) was identified.

**Fig. 1:**
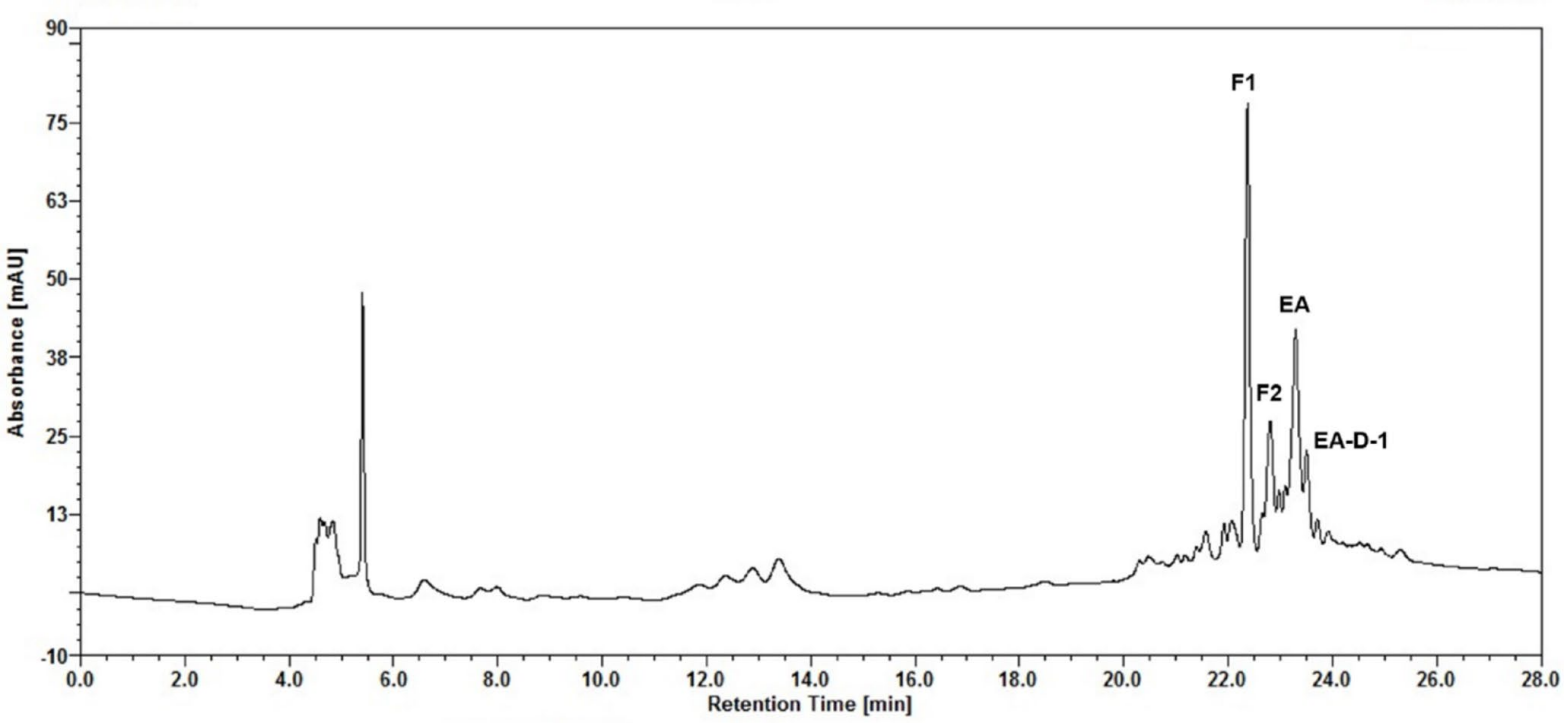
Chromatogram of the aqueous extract of *Psidium glaziovianum* leaves obtained at 270 nm. F1—Flavonoid 1; F2—Flavonoid 2; EA—Ellagic acid; EA-D-1—Ellagic acid derivative 1

**Fig. 2:**
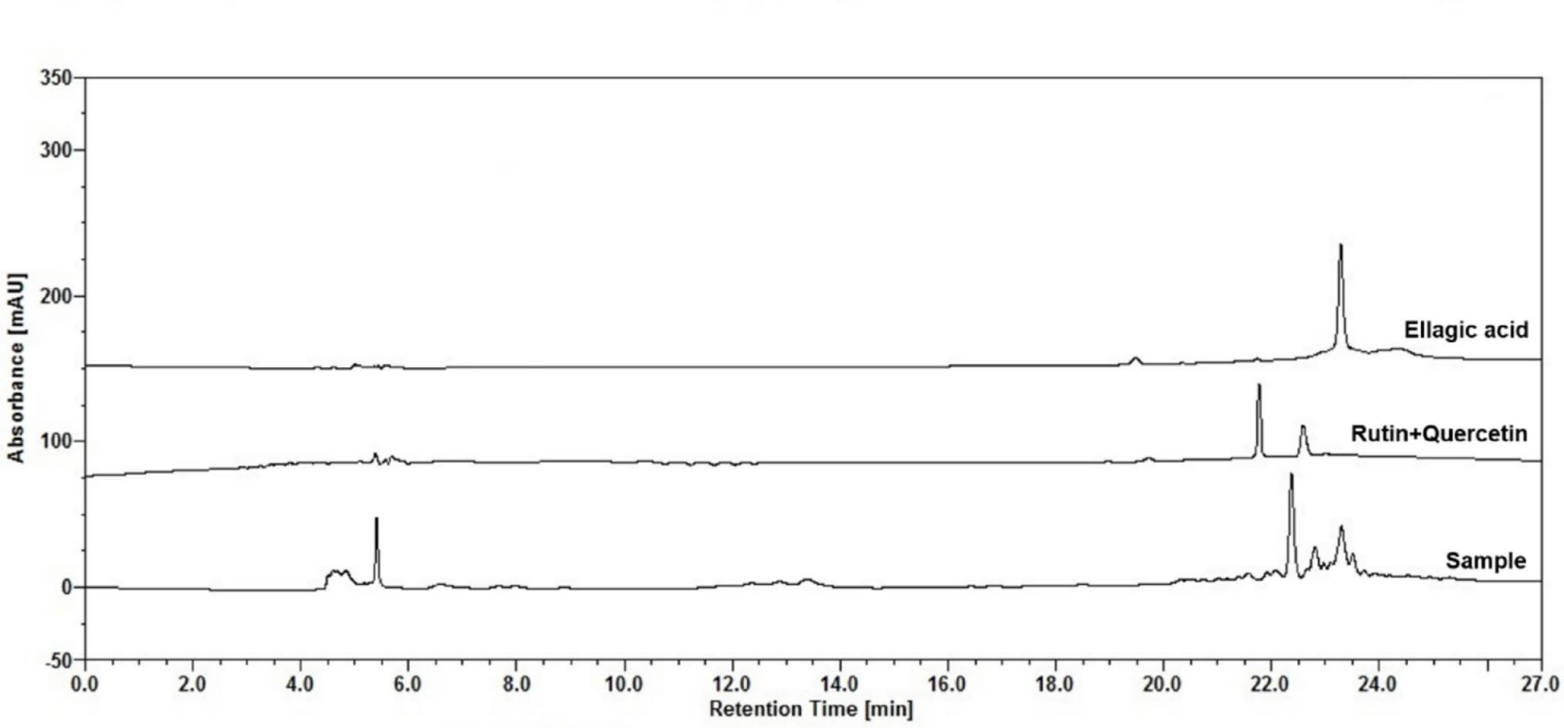
Comparative chromatogram obtained at 270 nm of the aqueous leaf extract of *Psidium glaziovianum* (bottom trace) and the reference standards ellagic acid (top trace) and rutin + quercetin mixture (middle trace)

**Fig. 3:**
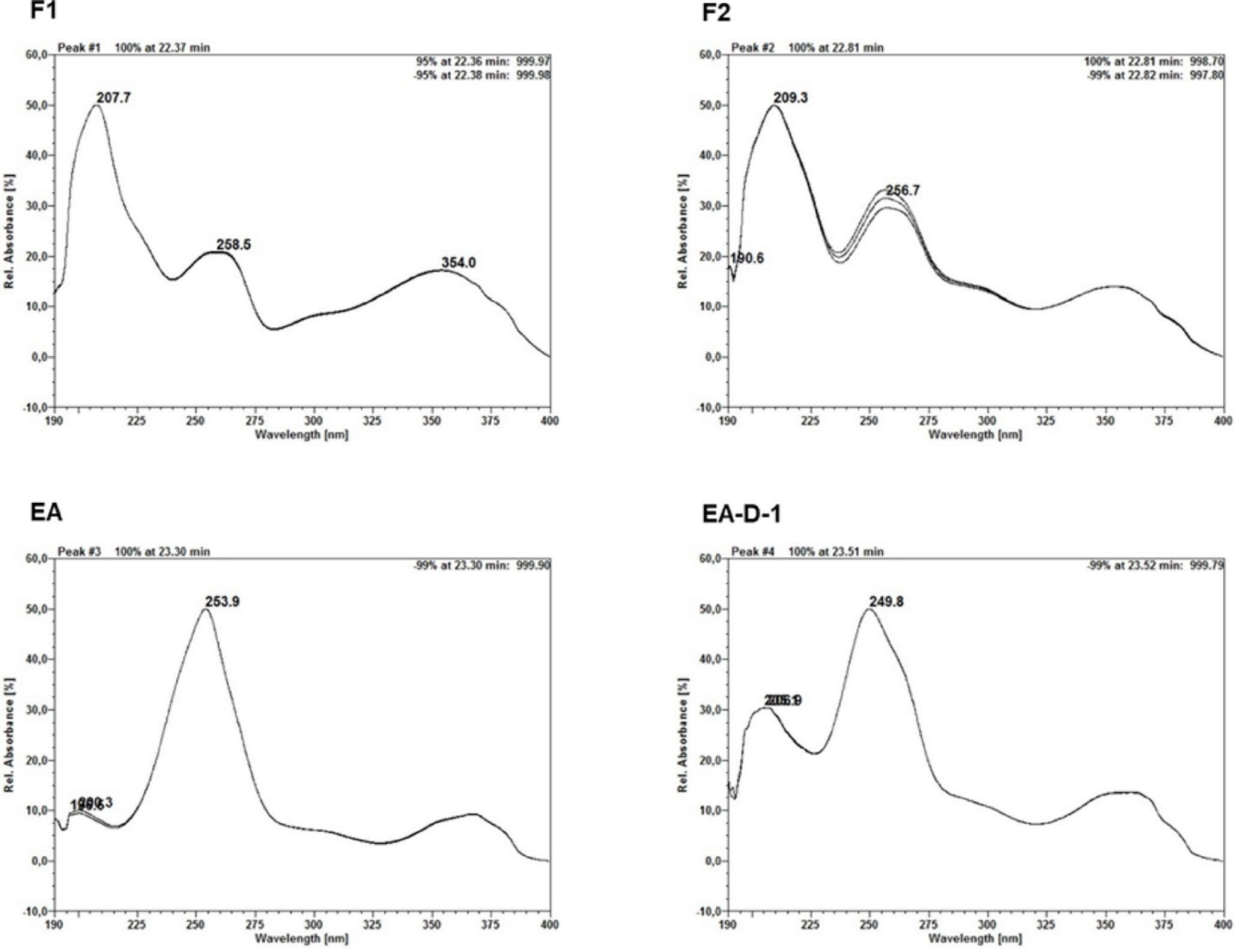
UV-Vis scan spectra of the peaks detected in the chromatogram of the aqueous leaf extract of *Psidium glaziovianum*. F1 – Flavonoid 1; F2 – Flavonoid 2; EA – Ellagic acid; EA-D-1 – Ellagic acid derivative 1

The chromatogram of AEPg revealed multiple peaks between approximately 20 and 25 min of retention time, with major peaks close to those observed for ellagic acid (~ 23.5 min) and rutin/quercetin (~ 22–23 min) standards. These peaks suggest the presence of phenolic compounds and flavonoids in the sample, although differences in retention time and intensity profiles indicate that the flavonoids in the extract are not identical to rutin or quercetin (Fig. 2). Nevertheless, they exhibit spectral similarity, as confirmed by the scan spectra (Fig. 3).

The overlap with the ellagic acid standard confirms the presence of this compound in the extract, identifying it as one of its major constituents. These findings support the polyphenolic composition of the extract and help explain its biological activity, as further investigated in the in vivo models.

The absorption spectra of the chromatographic peaks attributed to flavonoids and phenolic acids were analyzed. Flavonoid F1 showed absorption maxima at 207.7, 258.5, and 354.0 nm, while F2 presented maxima at 209.3, 256.7, and within the 300–370 nm range. These patterns are consistent with the characteristic structure of flavonoids, although they do not overlap with the spectra of rutin or quercetin standards, suggesting the presence of distinct flavonoids within the same class.

The peak identified as ellagic acid (EA) exhibited maxima at 198.3 and 253.9 nm, consistent with this phenolic compound. In turn, the ellagic acid derivative (EA-D-1) displayed maxima at 205.9 and 249.8 nm, confirming structural similarity but with shifts indicative of substitutions in the core structure. Spectral analysis therefore confirms the presence of polyphenolic compounds with features compatible with flavonoids and phenolic acids, corroborating the chromatographic data (Figs. 1 and 2) and contributing to the chemical characterization of the evaluated extract.

Quantitative analysis of the compounds identified by chromatography was performed using calibration curves of rutin (≥ 94%) and ellagic acid (≥ 95%), both obtained from Sigma-Aldrich^®^. Results are expressed as g% (mass of compound per 100 g of dry leaves) and presented as mean ± relative standard deviation, obtained in triplicate. Among the identified flavonoids, F1 exhibited the highest content at 2.14 g% (0.52%), followed by F2 at 0.54 g% (0.34%), both calculated as rutin equivalents. Ellagic acid (EA) was quantified at 0.49 g% (0.17%) and its derivative (EA-D-1) at 0.38 g% (0.04%), both expressed as ellagic acid equivalents.

These data confirm that flavonoids constitute the predominant fraction among the phenolic compounds in the extract, with F1 being the most abundant. The significant presence of ellagic acid and its derivatives is also consistent with the observed spectrophotometric profile (Fig. 3) and supports the rationale for the pharmacological properties evaluated in the in vivo models.

**Table 1:** Quantification of the major compounds in the aqueous leaf extract of *Psidium glaziovianum*

Compound	g% (mass of compound per 100 g of dry leaf)
F1^#^	2.14 g% (0.52%)
F2^#^	0.54 g% (0.34%)
EA	0.49 g% (0.17%)
EA-D-1*	0.38 g% (0.04%)

*F*1 Flavonoid 1, *F*2 Flavonoid 2, *EA* Ellagic acid, *EA*-*D*-1 Ellagic acid derivative 1. ^#^Calculated as rutin; *Calculated as ellagic acid. Results were obtained in triplicate and expressed as mean (relative standard deviation)

Flavonoids and ellagic acid are phenolic compounds widely distributed in plants and extensively studied for their pharmacological properties. They exhibit potent antioxidant activity, protecting cellular structures by neutralizing reactive oxygen and nitrogen species. They also display significant anti-inflammatory effects, mediated by the inhibition of enzymes such as COX and LOX, and by modulation of pro-inflammatory cytokine expression, including TNF-α, IL-1β, and IL-6. Additionally, they demonstrate antitumor effects through induction of apoptosis, inhibition of cell proliferation, and suppression of angiogenesis, generally associated with modulation of cellular signaling pathways (Gupta et al. 2021; Sharifi-Rad et al. 2022; Naraki et al. 2023; Lee et al. 2023; Li et al. 2023a, b).

In the cardiovascular system, these compounds support endothelial integrity, reduce LDL oxidation, inhibit platelet aggregation, and promote vasodilation, contributing to the prevention of atherosclerotic diseases. Studies also indicate neuroprotective activity, particularly in models of neurodegenerative disorders, due to their capacity to reduce inflammation in the central nervous system and protect neurons (Wang et al. 2022; Martínez-Coria et al. 2023).

Ellagic acid possesses strong antioxidant activity but is also notable for its anticancer properties, inhibiting tumor growth, blocking the cell cycle, inducing apoptosis, and positively modulating tumor suppressor genes. Moreover, it exerts anti-inflammatory, hepatoprotective, antimicrobial, and antidiabetic effects, improving insulin sensitivity, reducing glycemic levels, and attenuating diabetes-associated inflammatory processes. Together, these phenolic compounds are promising candidates for the development of natural therapeutic agents with multiple applications in the prevention and treatment of chronic and degenerative diseases (Chen et al. 2023; Billowria et al. 2024; Wenbo et al. 2024).

Following chemical characterization, the aqueous leaf extract of *P. glaziovianum* (AEPg) was evaluated pharmacologically. In the acetic acid-induced writhing model, treatment with AEPg resulted in a significant reduction (*p* < 0.001) in the number of writhes compared to the control group. A dose-dependent decrease in writhing was observed in extract-treated groups. The control group exhibited the highest number of writhes, demonstrating high nociceptive sensitivity to acetic acid. In contrast, groups treated with AEPg at 25, 50, and 100 mg/kg showed reductions of 20%, 50%, and 58.75%, respectively. As reference drugs, morphine (10 mg/kg) and indomethacin (20 mg/kg) produced reductions of 96.67% and 90%, respectively, in the number of writhes.

**Fig. 4:**
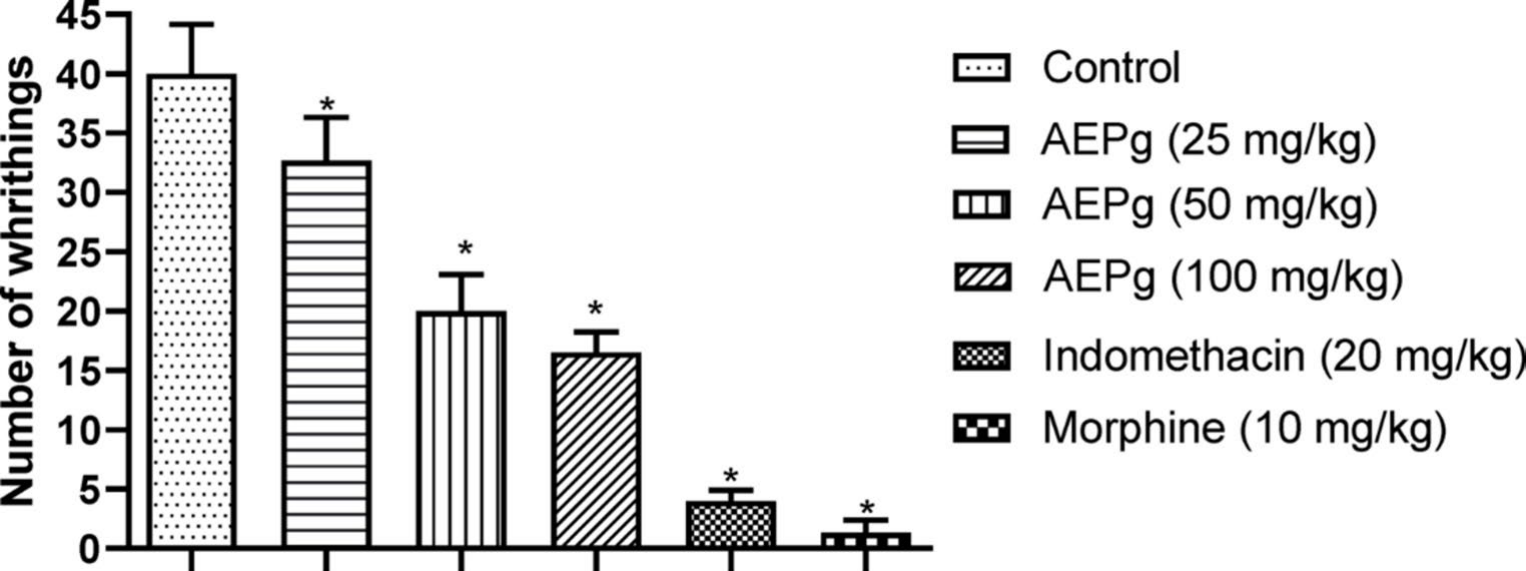
Antinociceptive effect of the aqueous extract of *P. glaziovianum* (AEPg) in the acetic acid-induced abdominal writhing model in mice. Values represent the mean ± SEM. *p < 0.001 compared with control, one-way analysis of variance (ANOVA) followed by Dunnett’s test

These findings indicate that AEPg exhibits a relevant and dose-dependent antinociceptive effect. The efficacy observed, particularly at the highest dose, suggests that AEPg may act on both peripheral and central pain modulation mechanisms. This activity can be attributed to its flavonoid and ellagic acid constituents, which may reduce acetic acid-induced abdominal writhes through their anti-inflammatory and analgesic properties (Forouzanfar et al. 2023; Al-Hoshary and Zalzala 2023; Jafari Karegar et al. 2023).

Acetic acid induces the release of inflammatory mediators such as prostaglandins, bradykinin, and histamine, which activate nerve endings, causing pain and writhing. Flavonoids act by inhibiting cyclooxygenase, reducing prostaglandin production, decreasing inflammatory cytokines and nitric oxide, and exhibiting antioxidant activity that attenuates nociceptor sensitization (Jennings et al. 2024; Bondonno et al. 2024; Wang et al. 2024). Ellagic acid also exerts anti-inflammatory effects by inhibiting pathways such as NF-κB and decreasing the release of inflammatory mediators, in addition to demonstrating proven antinociceptive effects in animal models (Forouzanfar et al. 2023; Al-Hoshary and Zalzala 2023; Jafari Karegar et al. 2023). Thus, the reduction in writhing indicates that these compounds exert peripheral analgesic effects.

The similarity between the effects observed and those produced by standard drugs such as morphine and indomethacin highlight the therapeutic potential of the extract and supports the need for further investigations into its mechanisms of action and possible applications in pain management. These findings align with previous reports for other species of the genus, such as *P. cattleianum*, whose hydroalcoholic leaf extract demonstrated a strong peripheral analgesic activity at doses of 60–400 mg/kg (Alvarenga et al. 2013), without exhibiting central effects. The analgesic action of AEPg may be associated with the inhibition of prostaglandin synthesis and/or modulation of ion channels involved in pain transduction, including TRPA1 and TRPV1 mechanisms previously linked to *Psidium* extracts Zhang et al. 2025). The detection of flavonoids in PgAE, evidenced by characteristic absorption peaks at 202, 258, and 354 nm, further supports this hypothesis, as these compounds are well known for their capacity to inhibit the cyclooxygenase pathway and suppress the release of inflammatory mediators.

Additionally, in the formalin test, the aqueous leaf extract of *P. glaziovianum* (AEPg) demonstrated significant analgesic effects in both phases of the nociceptive response. In the initial phase (0–5 min), associated with direct nociceptor stimulation (neurogenic phase), a reduction in paw-licking time was observed at all tested doses. The control group displayed a high paw-licking time (121 s), reflecting intense neurogenic pain. Groups treated with AEPg at 25, 50, and 100 mg/kg showed significant reductions, with the most pronounced effects observed at 50 and 100 mg/kg (48.35% and 48.64% inhibition, respectively). Morphine (10 mg/kg), used as a positive control, produced 90.08% inhibition, while indomethacin (20 mg/kg) reduced paw-licking time by 31.27%, also demonstrating relevant analgesic activity in this phase.

**Fig. 5:**
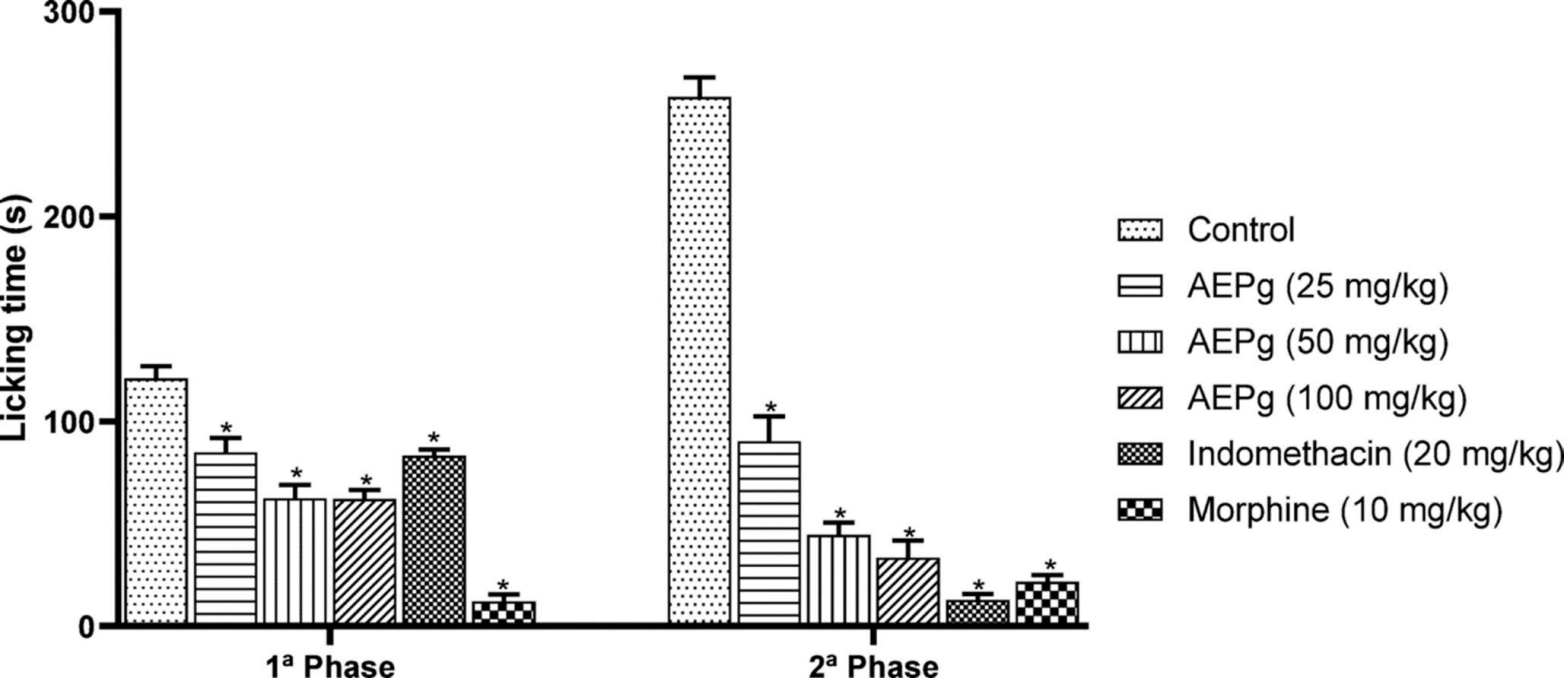
Antinociceptive effect of the aqueous extract of *P. glaziovianum* (AEPg) in the neurogenic and inflammatory phases of the formalin test in mice. Values represent the mean ± SEM. *p < 0.001 compared with control, two-way analysis of variance (ANOVA) followed by Dunnett’s test

In the second phase (15–30 min), characterized by an inflammatory process, the control group exhibited increased paw-licking time (258 s), indicating intense inflammatory pain. Treatment with AEPg at 25, 50, and 100 mg/kg significantly inhibited this response, with reductions of 65.04%, 82.79%, and 87.02%, respectively. These effects were comparable to those observed with indomethacin (95.10%) and morphine (91.61%). These results suggest that AEPg possesses potent anti-inflammatory activity and is capable of modulating both peripheral and central components of nociception (Figs. 4 and 5).

Formalin injection induces a biphasic pain response: an initial phase, caused by direct activation of peripheral nociceptors, and a late phase, associated with inflammation and the release of inflammatory mediators in the tissue (Rahimi et al. 2023; Saurin et al. 2025). Inhibition of the first phase suggests that the extract may directly interfere with nociceptor activation or modulate central pain pathways, possibly involving opioid receptors or ion channels. Significant inhibition of the second phase, particularly at doses of 50 and 100 mg/kg, indicates a clear anti-inflammatory effect, comparable to that of indomethacin. Flavonoids act by inhibiting cyclooxygenase (COX) and reducing the production of prostaglandins, inflammatory cytokines, and nitric oxide, thereby decreasing inflammation and nociceptor sensitization (Jennings et al. 2024; Bondonno et al. 2024; Wang et al. 2024).

These findings are consistent with the effects reported for *P. guineense*, whose hydromethanolic extract also exhibited antinociceptive activity in both phases of the test (Nascimento et al. 2021). However, unlike *P. cattleianum*, whose action is predominantly peripheral (Alvarenga et al. 2013). *P. glaziovianum* showed involvement of central mechanisms, suggesting the participation of muscarinic and opioid receptors. The combination of these mechanisms makes AEPg a promising candidate for the management of painful conditions involving both peripheral sensitization and central pain processing.

In the tail immersion test in hot water, a significant increase in withdrawal latency was observed in animals treated with AEPg compared to the control group. From 30 min onward, the latency of response was significantly increased in all AEPg-treated groups and in the morphine group (10 mg/kg) compared to control. This analgesic effect persisted over time, remaining until 120 min after administration. The 100 mg/kg dose of AEPg produced the longest latency times, approaching those observed in the morphine-treated group, indicating a more pronounced effect.

**Fig. 6:**
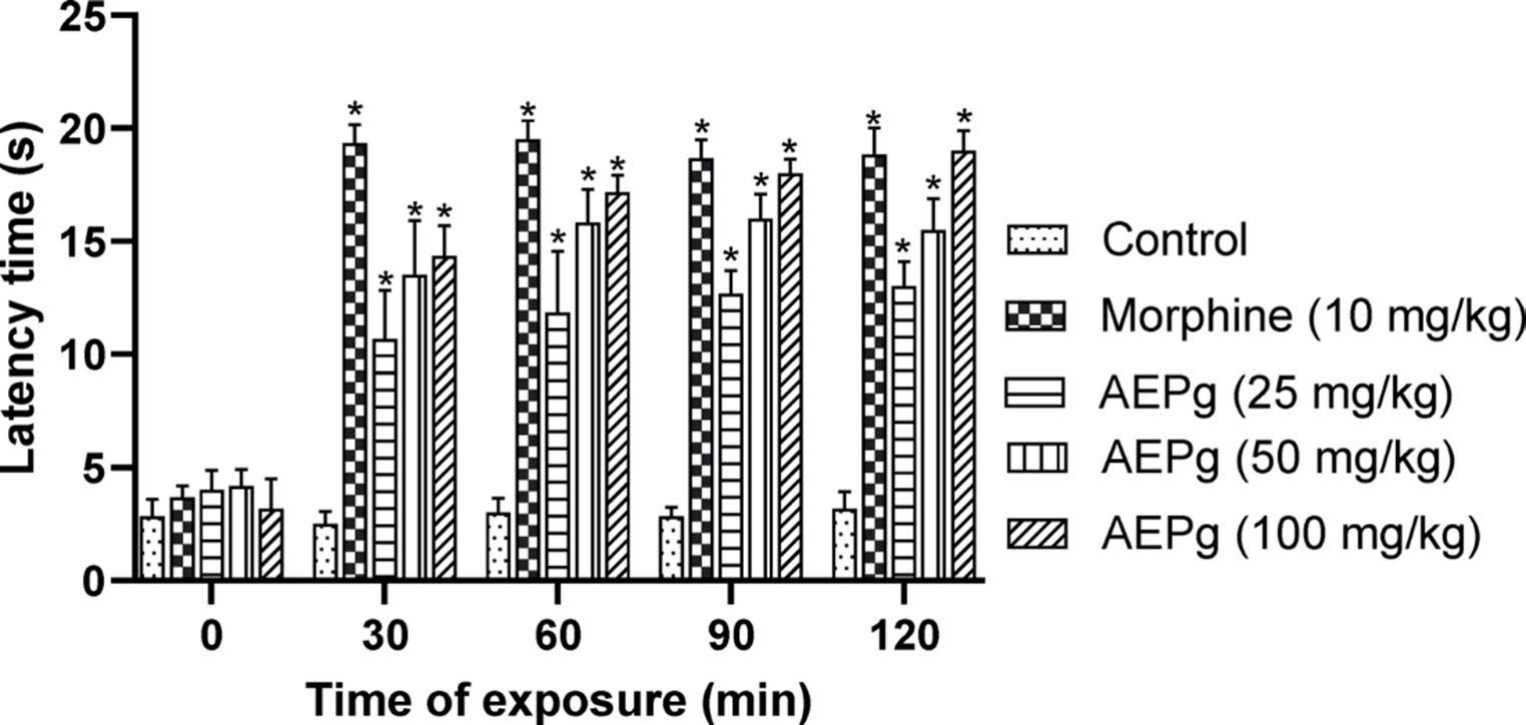
Effect of the aqueous extract of *P. glaziovianum* (AEPg) on response latency in the tail immersion test in mice. Values represent the mean ± SEM. *p < 0.001 compared with control, two-way analysis of variance (ANOVA) followed by Dunnett’s test

The results demonstrate that AEPg exhibits central antinociceptive activity, as it significantly increased response latency in the tail immersion test. This model is sensitive to opioid analgesics, such as morphine, and the effect observed with AEPg suggests that the extract may act on central pain modulation mechanisms, possibly involving opioid receptors or other central inhibitory pathways (Khatun et al. 2015; Oliveira et al. 2018).

Similar results were reported for the essential oil of *P. glaziovianum*, which also prolonged reaction time in thermal stimulus models (Costa et al. 2022), confirming the central action of this species. Comparatively, *P. guineense* showed a moderate effect in the same model (Akhter et al. 2021), while *P. cattleianum* did not demonstrate significant activity (Alvarenga et al. 2013). Thus, AEPg exerts broad analgesic effects involving both peripheral and central mechanisms.

To elucidate the mechanisms involved in the antinociceptive activity of AEPg, mice were pretreated with different pharmacological antagonists. Pretreatment with naloxone (2 mg/kg, i.p.), an opioid receptor antagonist, partially reversed the activity of AEPg, resulting in a paw-licking time of 102 ± 6.7 s, indicating the involvement of opioid receptors in the observed effect (*p* < 0.05 vs. AEPg). Similarly, cholinergic blockade with atropine (1 mg/kg, i.p.) also significantly inhibited the antinociceptive effect of AEPg (paw-licking time: 98 ± 5.9 s; *p* < 0.05 vs. AEPg), suggesting the participation of muscarinic receptors.

**Fig. 7:**
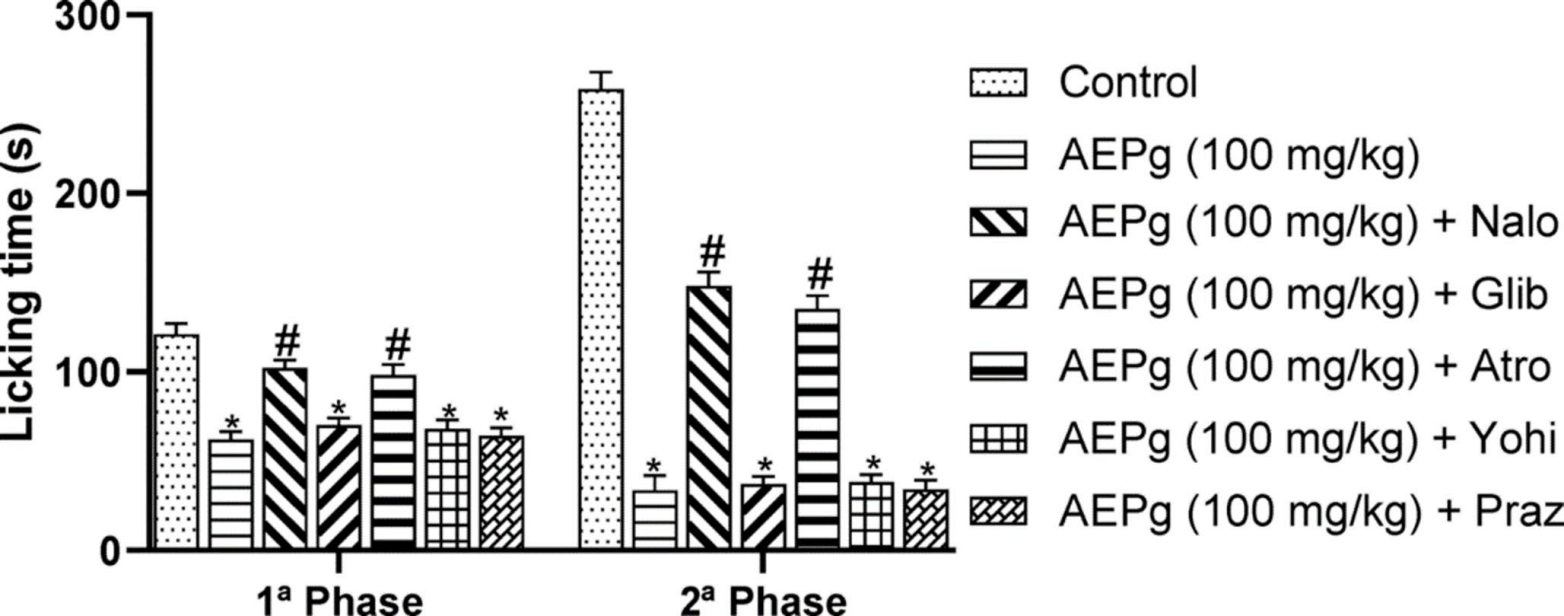
Effect of the aqueous leaf extract of *Psidium glaziovianum* (AEPg) on paw-licking time in both phases of the formalin test in mice, in the presence or absence of pharmacological antagonists

**Fig. 8:**
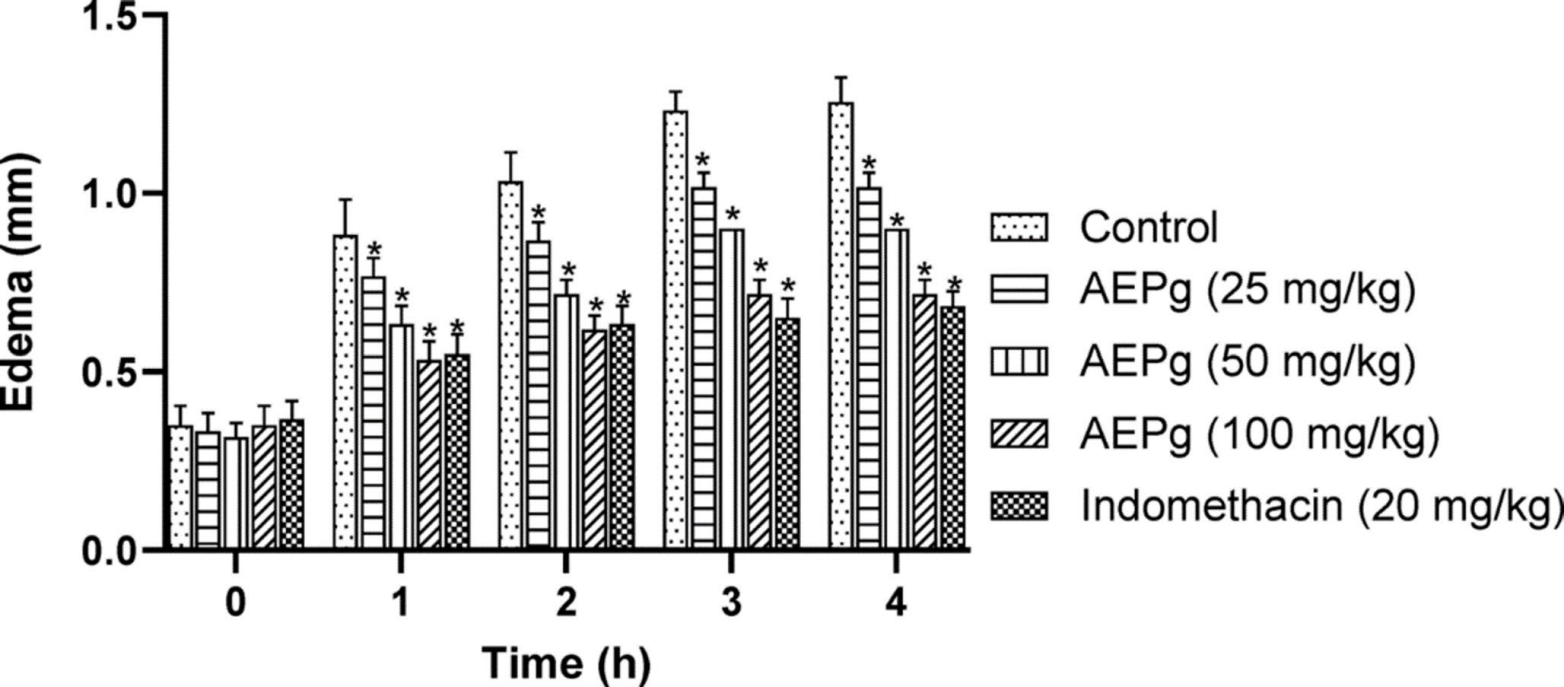
Effect of the aqueous extract of *P. glaziovianum* (AEPg) on carrageenan-induced paw edema in mice, Values represent the mean ± SEM. *p < 0.001 compared with control, two-way analysis of variance (ANOVA) followed by Dunnett’s test

In the second phase of the formalin test (15–30 min), the control group exhibited a mean paw-licking time of 258 ± 8.2 s. Both naloxone and atropine partially reversed this inhibition (paw-licking times of 148 ± 7.4 s and 135 ± 6.8 s, respectively; *p* < 0.05 vs. AEPg), reinforcing the involvement of opioid and muscarinic receptors in the modulation of the inflammatory response. In contrast, pretreatment with glibenclamide (K⁺(ATP) channel blocker), prazosin (α1-adrenergic antagonist), or yohimbine (α2-adrenergic antagonist) did not significantly alter the antinociceptive response promoted by AEPg in any phase, indicating that these systems are not directly involved in the extract’s activity (Figs. 6, 7 and 8).

The animals were pre-treated with naloxone (Nalox, 2 mg/kg, i.p.), glibenclamide (Glib, 5 mg/kg, i.p.), atropine (Atro, 1 mg/kg, i.p.), yohimbine (Yohim, 1 mg/kg, i.p.), or prazosin (Praz, 1 mg/kg, i.p.) 15 min before EAPg administration. Data are expressed as mean ± standard error of the mean (SEM) of licking time (in seconds), *n* = 6 per group. # *p* < 0.001 compared to the control group (saline); * *p* < 0.001 compared to the group treated with EAPg (100 mg/kg), according to ANOVA followed by Dunnett’s test.

The results suggest that compounds present in the extract may modulate or mimic the action of endogenous opioids, potentially acting on µ, κ, or δ receptors (Li et al. 2023a, b; Che and Roth 2023). This indicates a relevant analgesic potential, with the advantage of possibly exhibiting a more favorable safety profile compared to classical opioids, whose clinical use is limited by adverse effects and risk of dependence (Coates and Lazarus 2023; Nylander et al. 2024). Moreover, the inhibition of the extract’s antinociceptive activity by atropine points to the involvement of muscarinic receptors, reinforcing the role of the cholinergic system in pain modulation (Opretzka et al. 2023; Shen et al. 2024). The concurrent action on opioid and cholinergic pathways suggests a multimodal mechanism, broadening the therapeutic prospects of the extract.

The antinociceptive activity of AEPg observed in this study, mediated by opioid and muscarinic receptors, aligns with evidence reported for other species of the *Psidium* genus. Hydroalcoholic leaf extracts of *P. cattleianum* have shown peripheral analgesic activity (Alvarenga et al. 2013), while essential oil from *P. brownianum* leaves exerts analgesic effects via similar mechanisms involving the opioid system (Sampaio et al. 2020). Essential oil from *P. cattleyanum* leaves demonstrates analgesic and anti-inflammatory actions mediated by inhibition of prostaglandins and pro-inflammatory cytokines (Guimarães Silva et al. 2023).

This similarity suggests that bioactive compounds present in different *Psidium* species, such as terpenes, flavonoids, tannins, and phenolic acids, may share common pharmacological targets. Comparison with *P. glaziovianum* indicates that, although the species belong to the same genus, the pharmacological profile may vary regarding the extent of involvement of different pathways, highlighting the importance of species-specific investigations.

The reduction in acetic acid-induced abdominal writhing and the decrease in paw-licking time during the second phase of the formalin test after AEPg treatment indicate an anti-inflammatory effect. Therefore, the anti-inflammatory activity of AEPg was evaluated using two widely employed experimental models: carrageenan-induced paw edema and peritonitis.

The carrageenan-induced paw edema model in mice is one of the most commonly used experimental methods to assess the anti-inflammatory potential of natural and synthetic substances. Carrageenan injection elicits a localized inflammatory response characterized by edema, increased vascular permeability, and infiltration of inflammatory cells (Guihon et al. 2011).

Administration of AEPg produced a significant reduction in carrageenan-induced paw edema compared to the control group. The effect was dose-dependent, with greater inhibition observed at higher extract doses. In the first hour after induction, animals treated with AEPg showed a marked decrease in paw thickness, and this effect persisted over subsequent hours, indicating interference with both the early phase (mediated by histamine and serotonin) and the late phase (mainly mediated by prostaglandins and leukotrienes) of the inflammatory process. The group treated with the reference drug, indomethacin, showed reductions comparable to those observed with the highest doses of AEPg, suggesting that the extract has an anti-inflammatory profile similar to a classical non-steroidal anti-inflammatory drug.

The results obtained demonstrate that AEPg exerted a significant inhibitory effect on carrageenan-induced paw edema, an effect likely associated with the presence of phenolic compounds such as flavonoids and ellagic acid. These secondary metabolites are well-known for their antioxidant and anti-inflammatory activities, acting through multiple mechanisms, including the neutralization of reactive oxygen species, modulation of pro-inflammatory cytokine expression (TNF-α, IL-1β, IL-6), and inhibition of cyclooxygenase and lipoxygenase enzymes, thereby reducing the production of prostaglandins and leukotrienes. Additionally, flavonoids exert stabilizing effects on mast cells and capillaries, decreasing the release of histamine and serotonin and consequently reducing vascular permeability and cellular infiltration, contributing to the attenuation of both the early and late phases of inflammation (Tang et al. 2017; Yi 2018; Gupta et al. 2021; Sharifi-Rad et al. 2022; Naraki et al. 2023; Lee et al. 2023; Li et al. 2023a, b; Jamshidi et al. 2024).

Previous studies corroborate the findings of the present work by demonstrating that species of the *Psidium* genus exhibit anti-inflammatory activity in paw edema models. *P. guineense* extract significantly reduced edema in rodents, attributed to its high flavonoid and tannin content (Nascimento et al. 2021). Similarly, *P. guajava* displayed anti-edematogenic effects associated with phenolic compounds (Vasconcelos et al. 2017). Moreover, essential oils from *P. brownianum* (Souza Sampaio et al. 2024), P. *glaziovianum* (Costa et al. 2022)d *cattleyanum* also showed similar results, suggesting that this activity may be a common feature among species of the genus, likely due to their rich content of bioactive metabolites. These results reinforce the potential of AEPg as a source of pharmacologically relevant molecules capable of modulating central events of the acute inflammatory response.

The carrageenan-induced peritonitis model in mice is widely used to investigate the anti-inflammatory potential of natural and synthetic substances. Following intraperitoneal administration of carrageenan, there is recruitment of inflammatory cells, primarily neutrophils, as well as the release of pro-inflammatory mediators such as cytokines and chemokines. This model allows the evaluation of both the inhibition of cellular migration and the modulation of soluble inflammatory mediators in the peritoneal exudate, such as TNF-α and IL-1β, which play central roles in amplifying the acute inflammatory response (Nascimento et al. 2022; Costa et al. 2022).

The anti-inflammatory effect of AEPg treatment was evaluated using the carrageenan-induced peritonitis model, revealing significant changes in total leukocyte and neutrophil counts in the peritoneal fluid. Animals treated with AEPg at doses of 25, 50, and 100 mg/kg exhibited reductions of 35.80%, 55.55%, and 70.37% in total leukocyte counts, respectively, compared to the control group. Specifically, neutrophils decreased by 32.07%, 54.71%, and 67.92% at the same concentrations.

Analysis of pro-inflammatory cytokines showed that TNF-α levels in the peritoneal fluid decreased by 28.6%, 48.4%, and 61.6% in the groups treated with AEPg (25, 50, and 100 mg/kg), while IL-1β levels were reduced by 26.28%, 49.2%, and 61.4%, respectively, relative to the control. These results indicate a dose-dependent response to AEPg, reflecting its capacity to modulate cellular inflammation and soluble mediators during acute peritonitis. In comparison, indomethacin (20 mg/kg) reduced TNF-α by 64.8% and IL-1β by 66.2% (Tables 1 and 2).

**Table 2:** Effect of the aqueous extract of *P. glaziovianum* (AEPg) on total leukocytes, neutrophils, TNF-α, and IL-1β in carrageenan-induced peritonitis in mice

	Dose (mg/kg)	Leukocytes (10^5^/mL)	Neutrophils (10^5^/mL)	TNF-α (pg/mL)	IL-1β (pg/mL)
Vehicle	–	8.1 ± 0.9	5.3 ± 0.5	185.6 ± 12.4	142.8 ± 10.6
Indo	20	1.7 ± 0.6*	1.5 ± 0.3*	65.4 ± 8.1*	48.2 ± 6.9*
AEPg	25	5.2 ± 0.7*	3.6 ± 0.4*	132.5 ± 10.2*	105.4 ± 8.5*
AEPg	50	3.6 ± 0.5*	2.4 ± 0.3*	95.8 ± 7.4*	72.6 ± 6.2*
AEPg	100	2.4 ± 0.4*	1.7 ± 0.2*	71.3 ± 6.8*	53.7 ± 5.1*

Values represent the mean ± SEM. *p < 0.001 compared with control, one-way analysis of variance (ANOVA) followed by Dunnett’s test

The significant reduction in leukocyte recruitment and pro-inflammatory cytokine concentrations observed in the peritonitis model suggests that AEPg exerts a relevant anti-inflammatory effect by modulating the acute immune response. Previous studies with species of the *Psidium* genus, such as *P. guajava*, *P. cattleyanum*, and *P. glaziovianum*, also demonstrated anti-inflammatory effects in peritonitis models, highlighting the capacity of these extracts to inhibit neutrophil migration and reduce the production of inflammatory mediators (Costa et al. 2022; Shady et al. 2022; Guimarães Silva et al. 2023).

Phenolic compounds present in these extracts, particularly flavonoids and ellagic acid, have been implicated as responsible for these effects due to their antioxidant activity and their ability to inhibit pro-inflammatory enzymes and signaling pathways, including the downregulation of TNF-α and IL-1β production. The presence of these bioactive compounds in AEPg likely explains the dose-dependent effect observed in the reduction of cellular infiltration and cytokine levels in the peritoneal fluid. Overall, these findings reinforce the potential of AEPg as an anti-inflammatory agent in acute peritonitis (Gupta et al. 2021; Sharifi-Rad et al. 2022; Naraki et al. 2023; Lee et al. 2023; Li et al. 2023a, b).

Regarding safety considerations, although the present study did not include a dedicated toxicity evaluation of the aqueous extract, no signs of behavioral alterations, systemic toxicity, or mortality were observed at the tested doses. Furthermore, previous studies conducted by our research group demonstrated that the essential oil from *P. glaziovianum* leaves did not induce significant toxicological, biochemical, hematological, or histopathological alterations in acute and repeated-dose oral toxicity assays in Swiss mice at higher doses than those used in the present study (Costa et al. 2023; Kennedy Costa et al. 2023). While differences between essential oil and aqueous extract compositions must be considered, these findings support a preliminary safety profile for the species. Nevertheless, dedicated toxicological studies with the aqueous extract are warranted and are planned for future investigations.

## Conclusions

AEPg demonstrated the ability to modulate multiple pathways involved in pain perception, particularly the cholinergic and opioid systems, suggesting that its antinociceptive effects are mediated, at least in part, through interactions with muscarinic and opioid receptors that regulate both peripheral and central nociceptive transmission. In addition, AEPg exhibited significant anti-inflammatory activity, as evidenced by the reduction of paw edema, leukocyte recruitment, and pro-inflammatory cytokines (TNF-α and IL-1β) in the peritoneal fluid. These findings support an integrated pharmacological effect, in which the extract simultaneously interferes with nociceptive signaling and modulates key inflammatory mediators.

Taken together, the results highlight the therapeutic potential of AEPg as a natural bioactive source capable of targeting complex and interconnected physiological pathways associated with pain and inflammation, possibly with a lower risk of adverse effects compared to conventional drugs. Nevertheless, the study was conducted exclusively in male animals and did not include a comprehensive toxicological assessment of the aqueous extract. Future investigations should address sex-related biological differences, perform detailed safety evaluations, and further characterize the active constituents and receptor subtype-specific mechanisms underlying the observed pharmacological effects.

## Data Availability

No datasets were generated or analysed during the current study.
